# Di­chlorido­(4,4′-dimethyl-2,2′-bi­pyridine-κ^2^ *N*,*N*′)zinc(II) aceto­nitrile monosolvate

**DOI:** 10.1107/S241431462201149X

**Published:** 2022-12-06

**Authors:** Rafael A. Adrian, Jadan J. Rios, Hadi D. Arman

**Affiliations:** a Department of Chemistry and Biochemistry, University of the Incarnate Word, San Antonio, Texas 78209, USA; b Department of Chemistry, The University of Texas at San Antonio, San Antonio, Texas 78249, USA; Vienna University of Technology, Austria

**Keywords:** zinc, crystal structure, 4,4′-dimethyl-2,2′-bi­pyridine, 2,2′-bi-4-picoline, dmb, coordinating chloride, tetra­hedral coordination sphere

## Abstract

In the title complex, [ZnCl_2_(C_12_H_12_N_2_)]·CH_3_CN, the zinc(II) atom is fourfold coordinated by two chloride ligands and a bidentate 4,4′-dimethyl-2,2′-bi­pyridine ligand in a distorted tetra­hedral shape with a mol­ecule of aceto­nitrile sitting in the outer coordination sphere of the complex. π–π stacking inter­actions between the pyridyl rings in adjacent mol­ecules contribute to the alignment of the complexes in columns parallel to the *a* axis.

## Abstract 2

In the crystal structure of the title compound, the central zinc(II) atom is surrounded by a bidentate 4,4′-dimethyl-2,2′-bi­pyridine ligand and two coordinating chlorides in a distorted tetra­hedral shape with *π–π* stacking inter­actions contributing to the crystal packing.

## Graphical abstract

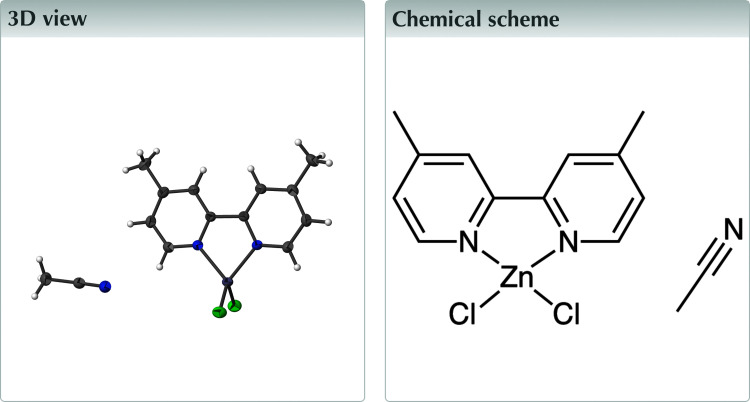

## Structure description

Over the last decade, metal complexes of 4,4′-dimethyl-2,2′-bi­pyridine have garnered significant attention due to their photophysical properties (Tamer *et al.*, 2020 ▸; Queiroz *et al.*, 2022 ▸), electrocatalytic activity (Ogihara *et al.*, 2018 ▸; Taylor *et al.*, 2018 ▸), and potential as anti­tumor agents (Amani *et al.*, 2014 ▸). Recently, platinum complexes incorporating 4,4′-dimethyl-2,2′-bi­pyridine were found to be effective against several cancer cell lines, including L1210 murine leukemia, HT29 human colon carcinoma, and U87 human glioblastoma (Pages *et al.*, 2015 ▸). Our research group inter­est currently lies in synthesizing metal complexes with applications in biological systems; as part of our research in this area, herein, we describe the synthesis and structure of the title complex, which promises to be a useful starting material in the synthesis of novel zinc(II) complexes.

**Figure 1:**
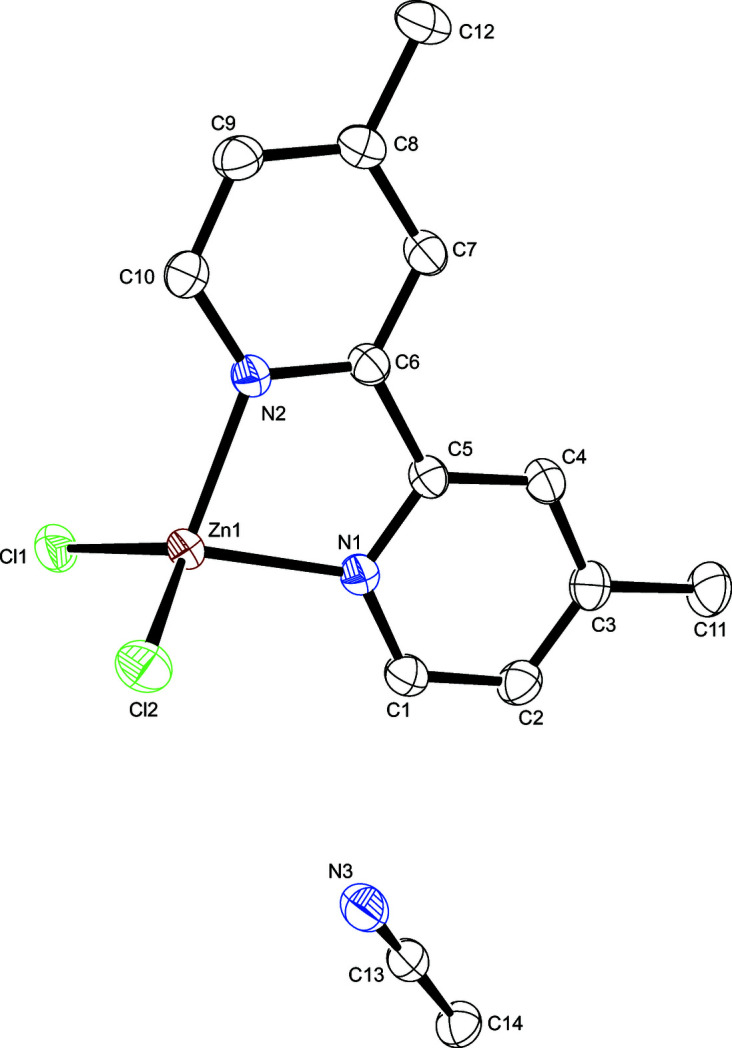
The structures of the mol­ecular entities of the title compound with displacement ellipsoids drawn at the 50% probability level; H atoms are omitted for clarity.

The asymmetric unit contains one mol­ecule of the title compound and one solvent mol­ecule of aceto­nitrile. The zinc(II) atom exhibits a distorted tetra­hedral cooordination environment defined by two pyridine nitro­gen atoms from the 4,4′-dimethyl-2,2′-bi­pyridine ligand and two chlorido ligands (Fig. 1 ▸). The Zn—N bond lengths are in good agreement with the comparable bromide analog complex currently available in the CSD (version 5.43 with update June 2022; Alizadeh *et al.*, 2010 ▸, refcode DURYAR) and with other 2,2′-bi­pyridine-based zinc(II) complexes (Khan & Tuck, 1984 ▸, refcode CEFFOI; Hossienifard *et al.*, 2011 ▸, refcode DAKMUZ; Nauha *et al.*, 2016 ▸, refcode EMERAR; Khalighi *et al.*, 2008 ▸, refcode POFKOL). Similar behavior is observed for the Zn—Cl bond lengths. The small bite angle N2—Zn1—N1 of 80.19 (7)° reflects the distortion from the ideal tetra­hedral coordination. Numerical data of relevant bonds and angles are presented in Table 1 ▸.

**Figure 2:**
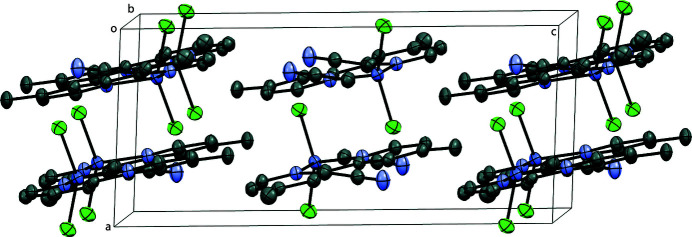
Perspective view of the crystal packing of the title complex approximately along the *b* axis; H atoms are omitted for clarity.

**Figure 3:**
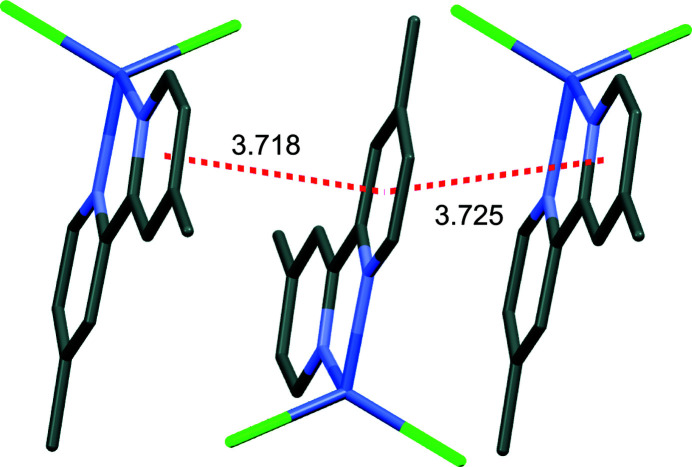
Capped sticks representation of the title mol­ecule showing *π–π* stacking inter­actions (red). H atoms and aceto­nitrile mol­ecule are omitted for clarity.

The title complex packs into layers extending parallel to the *bc* plane that are packed along the *a-*axis direction (Fig. 2 ▸). Contiguous pyridine rings show π–π stacking inter­actions, with centroid-to-centroid distances (*Cg*⋯*Cg*) alternating between 3.718 (1) Å and 3.725 (1) Å, and offset distances of 1.166 and 1.191 Å, respectively (Fig. 3 ▸). No other significant supra­molecular inter­action is present in the crystal packing of the title compound.

## Synthesis and crystallization

Zinc(II) chloride (0.370 g, 2.71 mmol) was added to a methanol solution (40 ml) of 4,4′-dimethyl-2,2′-bi­pyridine (0.500 g, 2.71 mmol). After stirring for 30 min, the resulting suspension was filtrated to obtain a white precipitate of the title compound (0.470 g, 54%). Crystals suitable for X-ray diffraction were obtained by vapor diffusion of diethyl ether over a saturated aceto­nitrile solution of the title compound at 277 K.

## Refinement

Crystal data, data collection and structure refinement details are summarized in Table 2 ▸.

## Supplementary Material

Crystal structure: contains datablock(s) I. DOI: 10.1107/S241431462201149X/wm4177sup1.cif

Structure factors: contains datablock(s) I. DOI: 10.1107/S241431462201149X/wm4177Isup2.hkl

Supporting information file. DOI: 10.1107/S241431462201149X/wm4177Isup3.mol

CCDC reference: 2223395

Additional supporting information:  crystallographic information; 3D view; checkCIF report

## Figures and Tables

**Table 1:** Selected geometric parameters (Å, °)

Zn1—Cl1	2.2065 (6)	Zn1—N1	2.0570 (17)
Zn1—Cl2	2.2005 (6)	Zn1—N2	2.0562 (17)
			
Cl2—Zn1—Cl1	116.82 (2)	N2—Zn1—Cl1	110.49 (5)
N1—Zn1—Cl1	117.12 (5)	N2—Zn1—Cl2	117.59 (5)
N1—Zn1—Cl2	109.48 (5)	N2—Zn1—N1	80.19 (7)

**Table 2:** Experimental details

Crystal data	
Chemical formula	[ZnCl_2_(C_12_H_12_N_2_)]·C_2_H_3_N
*M*_r_	361.56
Crystal system, space group	Monoclinic, *P*2_1_/*c*
Temperature (K)	100
*a*, *b*, *c* (Å)	7.2893 (1), 13.3443 (2), 16.1667 (3)
β (°)	92.486 (2)
*V* (Å^3^)	1571.06 (4)
*Z*	4
Radiation type	Cu *K*α
μ (mm^−1^)	5.23
Crystal size (mm)	0.26 × 0.10 × 0.05

Data collection	
Diffractometer	XtaLAB Synergy, Dualflex, HyPix
Absorption correction	Gaussian (*CrysAlis PRO*; Rigaku OD, 2020 ▸)
*T*_min_, *T*_max_	0.467, 1.000
No. of measured, independent and observed [*I* > 2σ(*I*)] reflections	15078, 3137, 2851
*R*_int_	0.040
(sin θ/λ)_max_ (Å^−1^)	0.630

Refinement	
*R*[*F* ^2^ > 2σ(*F* ^2^)], *wR*(*F* ^2^), *S*	0.032, 0.089, 1.07
No. of reflections	3137
No. of parameters	184
H-atom treatment	H-atom parameters constrained
Δρ_max_, Δρ_min_ (e Å^−3^)	0.47, −0.75

Computer programs: *CrysAlis PRO* (Rigaku OD, 2020 ▸), *SHELXT* (Sheldrick, 2015*a* ▸), *SHELXL* (Sheldrick, 2015*b* ▸), and *OLEX2* (Dolomanov *et al.*, 2009 ▸).

## full crystallographic data

### Crystal data

**Table:**

[ZnCl_2_(C_12_H_12_N_2_)]·C_2_H_3_N	*F*(000) = 736
*M**_r_* = 361.56	*D*_x_ = 1.529 Mg m^−^^3^
Monoclinic, *P*2_1_/*c*	Cu *K*α radiation, λ = 1.54184 Å
*a* = 7.2893 (1) Å	Cell parameters from 7600 reflections
*b* = 13.3443 (2) Å	θ = 4.3–75.6°
*c* = 16.1667 (3) Å	µ = 5.23 mm^−^^1^
β = 92.486 (2)°	*T* = 100 K
*V* = 1571.06 (4) Å^3^	Plank, clear colourless
*Z* = 4	0.26 × 0.10 × 0.05 mm

### Data collection

**Table:**

XtaLAB Synergy, Dualflex, HyPix diffractometer	3137 independent reflections
Radiation source: micro-focus sealed X-ray tube, PhotonJet (Cu) X-ray Source	2851 reflections with *I* > 2σ(*I*)
Mirror monochromator	*R*_int_ = 0.040
Detector resolution: 10.0000 pixels mm^-1^	θ_max_ = 76.3°, θ_min_ = 4.3°
ω scans	*h* = −9→8
Absorption correction: gaussian (CrysAlisPro; Rigaku OD, 2020)	*k* = −16→11
*T*_min_ = 0.467, *T*_max_ = 1.000	*l* = −20→18
15078 measured reflections	

### Refinement

**Table:**

Refinement on *F*^2^	Primary atom site location: dual
Least-squares matrix: full	Hydrogen site location: inferred from neighbouring sites
*R*[*F*^2^ > 2σ(*F*^2^)] = 0.032	H-atom parameters constrained
*wR*(*F*^2^) = 0.089	*w* = 1/[σ^2^(*F*_o_^2^) + (0.0506*P*)^2^ + 0.740*P*] where *P* = (*F*_o_^2^ + 2*F*_c_^2^)/3
*S* = 1.07	(Δ/σ)_max_ = 0.002
3137 reflections	Δρ_max_ = 0.47 e Å^−^^3^
184 parameters	Δρ_min_ = −0.75 e Å^−^^3^
0 restraints	

### Special details

**Table:**

Geometry. All esds (except the esd in the dihedral angle between two l.s. planes) are estimated using the full covariance matrix. The cell esds are taken into account individually in the estimation of esds in distances, angles and torsion angles; correlations between esds in cell parameters are only used when they are defined by crystal symmetry. An approximate (isotropic) treatment of cell esds is used for estimating esds involving l.s. planes.

### Fractional atomic coordinates and isotropic or equivalent isotropic displacement parameters (Å^2^)

**Table:**

	*x*	*y*	*z*	*U*_iso_*/*U*_eq_	
Zn1	0.26341 (4)	0.28826 (2)	0.57710 (2)	0.02312 (11)	
Cl1	0.52433 (7)	0.21775 (4)	0.61998 (3)	0.03091 (14)	
Cl2	0.01137 (8)	0.20050 (4)	0.59191 (4)	0.03469 (15)	
N1	0.2201 (2)	0.43399 (12)	0.61281 (10)	0.0234 (3)	
N2	0.2934 (2)	0.35984 (12)	0.46587 (10)	0.0238 (3)	
C6	0.2711 (3)	0.46056 (15)	0.46862 (12)	0.0229 (4)	
C5	0.2270 (3)	0.50205 (15)	0.55085 (12)	0.0224 (4)	
N3	0.2294 (3)	0.42938 (15)	0.88678 (12)	0.0386 (5)	
C4	0.1954 (3)	0.60303 (15)	0.56446 (13)	0.0257 (4)	
H4	0.199479	0.649436	0.520011	0.031*	
C7	0.2912 (3)	0.51951 (15)	0.39878 (13)	0.0258 (4)	
H7	0.275570	0.590064	0.401893	0.031*	
C1	0.1812 (3)	0.46558 (16)	0.68887 (13)	0.0271 (4)	
H1	0.174326	0.417554	0.731999	0.032*	
C8	0.3344 (3)	0.47496 (16)	0.32421 (13)	0.0271 (4)	
C2	0.1508 (3)	0.56532 (16)	0.70690 (13)	0.0287 (4)	
H2	0.125591	0.585262	0.761657	0.034*	
C10	0.3363 (3)	0.31691 (16)	0.39416 (13)	0.0277 (4)	
H10	0.352298	0.246295	0.392476	0.033*	
C3	0.1576 (3)	0.63621 (15)	0.64396 (13)	0.0279 (4)	
C9	0.3580 (3)	0.37158 (16)	0.32278 (13)	0.0287 (4)	
H9	0.388748	0.338853	0.273078	0.034*	
C13	0.2438 (3)	0.47359 (16)	0.94688 (14)	0.0296 (4)	
C12	0.3516 (3)	0.53683 (18)	0.24735 (14)	0.0350 (5)	
H12A	0.232760	0.539310	0.216670	0.053*	
H12B	0.443377	0.506603	0.212485	0.053*	
H12C	0.389982	0.604955	0.262723	0.053*	
C14	0.2648 (3)	0.52852 (18)	1.02426 (14)	0.0334 (5)	
H14A	0.321179	0.593786	1.014092	0.050*	
H14B	0.343360	0.490338	1.063596	0.050*	
H14C	0.143993	0.538499	1.047313	0.050*	
C11	0.1310 (3)	0.74589 (17)	0.66062 (16)	0.0360 (5)	
H11A	0.248128	0.781058	0.656074	0.054*	
H11B	0.086842	0.754766	0.716555	0.054*	
H11C	0.040579	0.773390	0.620108	0.054*	

### Atomic displacement parameters (Å^2^)

**Table:**

	*U*^11^	*U*^22^	*U*^33^	*U*^12^	*U*^13^	*U*^23^
Zn1	0.02849 (17)	0.01792 (16)	0.02321 (17)	0.00069 (9)	0.00410 (11)	0.00276 (9)
Cl1	0.0305 (3)	0.0271 (3)	0.0353 (3)	0.00510 (18)	0.0030 (2)	0.00574 (19)
Cl2	0.0334 (3)	0.0319 (3)	0.0392 (3)	−0.0078 (2)	0.0068 (2)	0.0031 (2)
N1	0.0274 (8)	0.0205 (8)	0.0223 (8)	0.0008 (6)	0.0021 (6)	0.0005 (6)
N2	0.0300 (8)	0.0187 (8)	0.0227 (8)	0.0000 (6)	0.0028 (6)	0.0006 (6)
C6	0.0253 (9)	0.0213 (9)	0.0221 (9)	−0.0014 (7)	0.0002 (7)	0.0011 (7)
C5	0.0235 (9)	0.0209 (9)	0.0225 (9)	0.0001 (7)	−0.0017 (7)	0.0013 (8)
N3	0.0588 (13)	0.0285 (10)	0.0286 (10)	−0.0025 (9)	0.0040 (9)	−0.0004 (8)
C4	0.0292 (10)	0.0215 (10)	0.0260 (10)	0.0001 (8)	−0.0014 (8)	0.0013 (8)
C7	0.0300 (10)	0.0226 (9)	0.0245 (10)	−0.0007 (8)	−0.0015 (8)	0.0028 (8)
C1	0.0322 (10)	0.0261 (10)	0.0229 (10)	0.0009 (8)	0.0013 (8)	0.0023 (8)
C8	0.0286 (10)	0.0297 (10)	0.0229 (10)	−0.0003 (8)	−0.0004 (8)	0.0038 (8)
C2	0.0318 (10)	0.0292 (11)	0.0253 (10)	0.0011 (8)	0.0021 (8)	−0.0046 (8)
C10	0.0336 (10)	0.0235 (10)	0.0262 (10)	−0.0001 (8)	0.0027 (8)	−0.0028 (8)
C3	0.0285 (10)	0.0233 (10)	0.0317 (11)	0.0003 (8)	−0.0012 (8)	−0.0033 (8)
C9	0.0329 (10)	0.0309 (11)	0.0224 (9)	−0.0009 (8)	0.0018 (8)	−0.0025 (8)
C13	0.0344 (11)	0.0267 (10)	0.0280 (11)	0.0008 (8)	0.0023 (8)	0.0025 (9)
C12	0.0434 (13)	0.0386 (12)	0.0229 (10)	0.0005 (10)	0.0010 (9)	0.0083 (9)
C14	0.0374 (12)	0.0341 (12)	0.0288 (11)	0.0012 (9)	0.0009 (9)	−0.0042 (9)
C11	0.0435 (13)	0.0233 (11)	0.0414 (13)	−0.0009 (9)	0.0024 (10)	−0.0075 (9)

### Geometric parameters (Å, º)

**Table:**

Zn1—Cl1	2.2065 (6)	C8—C9	1.390 (3)
Zn1—Cl2	2.2005 (6)	C8—C12	1.502 (3)
Zn1—N1	2.0570 (17)	C2—H2	0.9500
Zn1—N2	2.0562 (17)	C2—C3	1.392 (3)
N1—C5	1.355 (3)	C10—H10	0.9500
N1—C1	1.342 (3)	C10—C9	1.380 (3)
N2—C6	1.355 (3)	C3—C11	1.502 (3)
N2—C10	1.342 (3)	C9—H9	0.9500
C6—C5	1.488 (3)	C13—C14	1.452 (3)
C6—C7	1.389 (3)	C12—H12A	0.9800
C5—C4	1.386 (3)	C12—H12B	0.9800
N3—C13	1.138 (3)	C12—H12C	0.9800
C4—H4	0.9500	C14—H14A	0.9800
C4—C3	1.398 (3)	C14—H14B	0.9800
C7—H7	0.9500	C14—H14C	0.9800
C7—C8	1.392 (3)	C11—H11A	0.9800
C1—H1	0.9500	C11—H11B	0.9800
C1—C2	1.382 (3)	C11—H11C	0.9800
			
Cl2—Zn1—Cl1	116.82 (2)	C1—C2—C3	119.29 (19)
N1—Zn1—Cl1	117.12 (5)	C3—C2—H2	120.4
N1—Zn1—Cl2	109.48 (5)	N2—C10—H10	118.8
N2—Zn1—Cl1	110.49 (5)	N2—C10—C9	122.41 (19)
N2—Zn1—Cl2	117.59 (5)	C9—C10—H10	118.8
N2—Zn1—N1	80.19 (7)	C4—C3—C11	120.37 (19)
C5—N1—Zn1	114.53 (13)	C2—C3—C4	118.13 (19)
C1—N1—Zn1	126.54 (14)	C2—C3—C11	121.5 (2)
C1—N1—C5	118.89 (17)	C8—C9—H9	120.3
C6—N2—Zn1	114.51 (13)	C10—C9—C8	119.47 (19)
C10—N2—Zn1	126.48 (14)	C10—C9—H9	120.3
C10—N2—C6	118.99 (17)	N3—C13—C14	178.8 (3)
N2—C6—C5	115.42 (17)	C8—C12—H12A	109.5
N2—C6—C7	121.21 (18)	C8—C12—H12B	109.5
C7—C6—C5	123.36 (18)	C8—C12—H12C	109.5
N1—C5—C6	115.31 (17)	H12A—C12—H12B	109.5
N1—C5—C4	121.51 (18)	H12A—C12—H12C	109.5
C4—C5—C6	123.18 (18)	H12B—C12—H12C	109.5
C5—C4—H4	120.2	C13—C14—H14A	109.5
C5—C4—C3	119.63 (19)	C13—C14—H14B	109.5
C3—C4—H4	120.2	C13—C14—H14C	109.5
C6—C7—H7	120.1	H14A—C14—H14B	109.5
C6—C7—C8	119.85 (19)	H14A—C14—H14C	109.5
C8—C7—H7	120.1	H14B—C14—H14C	109.5
N1—C1—H1	118.7	C3—C11—H11A	109.5
N1—C1—C2	122.54 (19)	C3—C11—H11B	109.5
C2—C1—H1	118.7	C3—C11—H11C	109.5
C7—C8—C12	120.8 (2)	H11A—C11—H11B	109.5
C9—C8—C7	118.07 (19)	H11A—C11—H11C	109.5
C9—C8—C12	121.12 (19)	H11B—C11—H11C	109.5
C1—C2—H2	120.4		
			
Zn1—N1—C5—C6	−2.1 (2)	C6—C7—C8—C12	178.3 (2)
Zn1—N1—C5—C4	178.15 (15)	C5—N1—C1—C2	−1.1 (3)
Zn1—N1—C1—C2	−178.79 (15)	C5—C6—C7—C8	179.38 (19)
Zn1—N2—C6—C5	−0.7 (2)	C5—C4—C3—C2	−0.8 (3)
Zn1—N2—C6—C7	178.55 (15)	C5—C4—C3—C11	177.39 (19)
Zn1—N2—C10—C9	−178.35 (16)	C7—C6—C5—N1	−177.36 (19)
N1—C5—C4—C3	0.8 (3)	C7—C6—C5—C4	2.4 (3)
N1—C1—C2—C3	1.0 (3)	C7—C8—C9—C10	0.7 (3)
N2—C6—C5—N1	1.8 (3)	C1—N1—C5—C6	179.95 (18)
N2—C6—C5—C4	−178.40 (18)	C1—N1—C5—C4	0.2 (3)
N2—C6—C7—C8	0.2 (3)	C1—C2—C3—C4	−0.1 (3)
N2—C10—C9—C8	−0.2 (3)	C1—C2—C3—C11	−178.2 (2)
C6—N2—C10—C9	−0.3 (3)	C10—N2—C6—C5	−178.96 (18)
C6—C5—C4—C3	−179.00 (18)	C10—N2—C6—C7	0.2 (3)
C6—C7—C8—C9	−0.7 (3)	C12—C8—C9—C10	−178.3 (2)
